# Individuals With Food Addiction After Metabolic And Bariatric Surgery Show Higher Consumption of Ultra-Processed Foods, Sedentary Lifestyle, Anxiety, and Sub-Optimal Body Weight Trajectories

**DOI:** 10.1007/s11695-026-08812-0

**Published:** 2026-06-27

**Authors:** Maria Clara Farias Tavares da Silva, Jennifer Mikaella Ferreira Melo, Natália Gomes da Silva Lopes, André Eduardo da Silva Júnior, Mateus de Lima Macena, Nassib Bezerra Bueno

**Affiliations:** 1 Laboratório de Nutrição e Metabolismo (LANUM), Programa de Pós-Graduação em Nutrição (PPGNUT), Faculdade de Nutrição (FANUT), Universidade Federal de Alagoas (UFAL), AL Maceió, Brazil; 2 Programa de Pós-Graduação em Nutrição , Escola Paulista de Medicina, Universidade Federal de São Paulo, SP São Paulo, Brazil

**Keywords:** Food addiction, Ultra-processed foods, Anxiety, Recurrent weight gain

## Abstract

**Introduction:**

Food addiction (FA) may impact weight-loss maintenance and metabolic outcomes after metabolic and bariatric surgery (MBS). This study assessed the clinical, dietary, behavioral, and anthropometric profiles of women in the postoperative period of MBS, according to the presence of FA.

**Methods:**

A cross-sectional study of women ≥ 18 months post–MBS, with data collected via an online questionnaire. FA was assessed using the modified Yale Food Addiction Scale 2.0. Dietary intake was evaluated by the NOVA-UPF score and by dietary markers from the Brazilian Food and Nutrition Surveillance System. Anxiety was measured using the Generalized Anxiety Disorder 7 questionnaire. Multivariable models adjusted for confounders were used to test associations.

**Results:**

631 women were included, with a prevalence of FA of 19.7%. Women with FA exhibited higher anxiety frequency (69.4% vs. 32.5%, p < 0.001), lower engagement in physical activity (38.7% vs. 59.4%, p < 0.001), and higher consumption of ultra-processed foods (4.37 vs. 3.26, p < 0.001). The FA group had a lower percentage of total weight loss (TWL: 26.96% vs. 34.25%, p < 0.001) and a higher current BMI (31.75 kg/m² vs. 27.96 kg/m², p < 0.001).

**Conclusion:**

FA was associated with higher current BMI, lower %TWL, higher anxiety symptoms, higher consumption of UPF, and lower engagement in physical activity in women after MBS. These findings reflect associations rather than causal relationships and underscore the importance of continuous screening and multidisciplinary follow-up for women after surgery.

**Graphical Abstract:**

**Figure:**
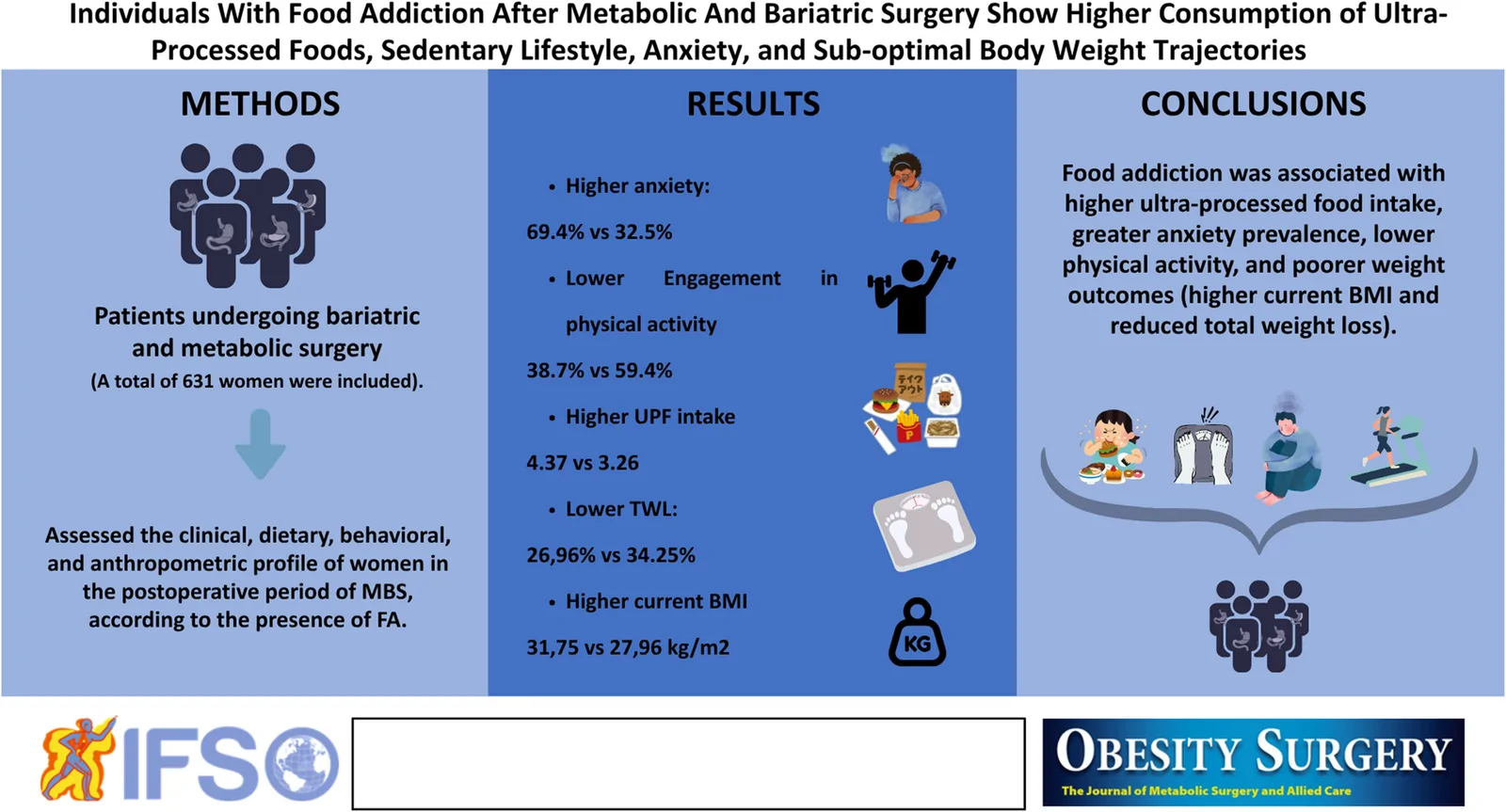

**Supplementary Information:**

The online version contains supplementary material available at https://doi.org/10.1007/s11695-026-08812-0.

## Key Points

Women with food addiction showed higher consumption of ultra-processed foods.

Food addiction was associated with a greater prevalence of anxiety and lower engagement in physical activity.

Participants with food addiction exhibited sub-optimal weight outcomes, including higher current BMI and lower total weight loss.

**Supplementary Information:**

The online version contains supplementary material available at https://doi.org/10.1007/s11695-026-08812-0.

## Introduction

It is estimated that a significant proportion of patients undergoing metabolic and bariatric surgery (MBS) experience recurrent weight gain of 10% or more of their body weight after a few years, a phenomenon associated with the resumption of inadequate eating behaviors, such as increased consumption of ultra-processed foods (UPFs) [1, 2], and with the absence of continuous follow-up by a multidisciplinary team [3]. Food addiction (FA) has been proposed as a potential factor contributing to this phenomenon, with its prevalence increasing significantly worldwide [4]. FA is characterized by compulsive consumption of highly palatable, energy-dense foods and shares features with substance use disorders [5]. FA, most commonly assessed using the Yale Food Addiction Scale (YFAS) and its subsequent versions, is frequently observed among candidates for MBS [6]. A systematic review found that the prevalence of FA reaches approximately 32% in the preoperative period and decreases to around 15% in the postoperative period [7].

Previous studies have reported associations between FA and lower percentage of total weight loss (%TWL) [8, 9]. For instance, Guerrero Pérez et al. [10] found that preoperative FA, assessed with a YFAS-based instrument, was linked to suboptimal preoperative weight loss in patients seeking MBS. In addition to weight outcomes, FA has been associated with higher prevalence of mood and anxiety disorders in this population [11]. In the postoperative context, Lobão et al. [2] investigated UPF consumption for 5 years after MBS. They found that dietary intake of such foods tends to return to the pre-surgical level after 60 months, even though they did not assess the role of FA in this intake. Mousavi et al. [8] evaluated the effects of FA two years after vertical sleeve gastrectomy, and found that individuals with FA presented lower physical activity levels than individuals without the FA; however, they did not measure UPF intake [8]. Finally, Cassin et al. [12] investigated food addiction, measured with the mYFAS 2.0, in relation to binge eating and symptoms of depression and anxiety, and tested a cognitive behavioural therapy intervention in post-operative MBS patients; however, they did not evaluate its association with UPF intake.

Given the complex interplay among FA, anxiety, sedentary behavior, UPF intake, and weight outcomes after MBS, it is important to examine these factors within the same sample. Therefore, this study aimed to characterize the clinical, dietary, behavioral, and anthropometric profiles of post-MBS individuals according to the presence of FA.

## Methods

### Ethical Aspects

This study was approved by the Research Ethics Committee of the Federal University of Alagoas (CAAE number: 60233722.7.0000.5013). All participants had access to the Informed Consent Form (ICF), which was available on the first page of the online questionnaire. Consent to participate was required to access the questionnaire and begin data collection. If the participant chose not to take part, the subsequent page displayed the message: “Thank you! You may close this page.” No personal information was collected from the participants.

### Study Design

This is a cross-sectional study, reported in accordance with the Strengthening the Reporting of Observational Studies in Epidemiology (STROBE) guidelines (Table S1) [13], and to the Checklist for Reporting Results of Internet E-Surveys (CHERRIES) (Table S2) [14]. Previous findings from this study are published in another report, and may be consulted for further information [15]. The present study utilizes a subsample of the main study sample.

### Procedures

After the research team developed and approved the research questionnaire, an electronic form was created using Google Forms. A pilot study was conducted with 20 participants. The average time to complete the questionnaire was 12 min and 46 s. In certain sections of the questionnaire, participants were instructed to select specific alternatives to monitor their attention while completing the task. The questionnaire consisted of 11 pages, each containing 6–10 items. The questions were presented in a fixed order, with no randomization or conditional branching, meaning that all participants responded to every item. Respondents could review and modify their answers before submission using the “back” button on the form. All responses submitted via the Google Forms^®^ link were automatically compiled into an online spreadsheet accessible exclusively to the research team. Due to the limitations of the Google Forms^®^ platform, view, participation, and completion rates could not be retrieved. Participant uniqueness was verified through the email address used to complete the form, with duplicate entries retaining only the most recent response. Only fully completed questionnaires were included in the analysis.

### Sampling and Eligibility Criteria

A non-probabilistic convenience sampling, snowballing method was employed. Invitation links were disseminated through the researchers’ social media profiles (Instagram and Facebook), as well as through channels related to MBS, whether managed by scientific societies or by health professionals involved in the care of these patients, in an “open-survey” fashion. Participant recruitment occurred between August 2022 and January 2023. Individuals of both sexes, aged between 18 and 59 years, who had undergone any technique of MBS were included. Pregnant and lactating women were excluded. For the present study, only individuals with pre-surgical BMI ≥ 30 kg/m² and at least 18 months post-surgery were included to avoid the short-term effects of surgery on FA. Anthropometric data were self-reported. Individuals who reported implausible post-surgical bodyweight values, defined as (1) post-surgical weight higher than preoperative baseline weight or (2) post-surgical weight higher than current bodyweight, were excluded. By definition, minimum weight should represent the lowest weight achieved after surgery; therefore, these records were excluded to avoid misclassification. These patterns were considered implausible because they preclude valid calculation of weight-loss trajectories (e.g., %TWL and nadir BMI) and likely reflect reporting or data entry errors. After applying eligibility and data-quality criteria, few men remained in the cohort (*n* = 22), and they were therefore excluded from inferential analyses due to insufficient statistical power and marked sex imbalance, in order to avoid unstable estimates. Participation in the survey was voluntary, and no incentives or compensation were offered.

### Exposure Variable

Food addiction: measured using the modified Yale Food Addiction Scale 2.0 (mYFAS 2.0). This scale consists of 13 items, 11 of which are related to the individual’s behavioral symptoms, based on the DSM-5 diagnostic criteria for substance-related use disorders. The remaining two items refer to the individual’s impairment and clinical distress. Individuals who presented two or more symptoms and who met the threshold for any of the impairment or clinical distress questions were classified as having FA. Participants were also classified according to addiction severity: no FA (one or no symptom), mild (two or three symptoms, with clinical significance), moderate (four or five symptoms, with clinical significance), and severe (six or more symptoms, with clinical significance) [15, 16].

### Outcomes

Generalized Anxiety Disorder: assessed through the GAD-7 questionnaire. This scale comprises seven items in a Likert format, with four response scores ranging from 0 to 3, where “0” corresponds to “not at all” and “3” to “nearly every day.” The sum of the scores assigned to the items yields a total score ranging from 0 to 21. Individuals who scored equal to or greater than 10 points were considered possible cases of GAD [17].

Regular physical activity: assessed through a single question asking whether the participant engages in at least 150 min of moderate physical exercise (dancing, household tasks, walking, etc.) or 75 min of vigorous physical exercise (swimming, cycling, playing sports, etc.) during the week.

Anthropometric variables: Participants self-reported their preoperative, lowest post-surgical, and current weight, as well as height. These data were self-reported, provided through the questions: “What is your current weight?”, “What was your weight before surgery?”, “What is the lowest weight you reached after surgery?” and “What is your height?”. From this, it was possible to assess ideal weight and body mass index (BMI) at three time points (preoperative, nadir, and current), as well as pre-surgical excess weight, defined as values above 25 kg/m² calculated from the preoperative BMI. The percentage of total weight loss (%TWL) was represented by the proportion of body weight reduction between the preoperative period and the current weight, with the initial weight used as the denominator and the result expressed as a percentage.

Dietary intake: consumption markers from the Brazilian Food and Nutrition Surveillance System (SISVAN) [18] were used. The instrument has nine items and assesses the frequency of daily meals, the habit of eating while watching screens, and the frequency of consumption of specific foods on the previous day. Furthermore, intake of ultra-processed foods (UPFs) was measured using the NOVA-UPF Screener, a self-administered questionnaire based on the NOVA classification [19]. The instrument comprises 23 food items, selected from the Household Budget Survey conducted by the Brazilian Institute of Geography and Statistics [20]. The score is obtained by summing the UPF subgroups consumed by the participant, ranging from 0 to 23 points.

Chronic diseases: Information on hypertension and diabetes mellitus was collected at a single time point, when participants completed the questionnaire, and was based on self-reported medical diagnosis. These variables, therefore, reflect the current diagnosis status at the time of data collection. Participants were asked the following questions: “Do you have a medical diagnosis of high blood pressure (hypertension)?” and “Do you have a medical diagnosis of diabetes?”. No information was collected regarding pre-surgical diagnosis status or post-surgical clinical outcomes, such as disease remission or persistence.

### Statistical Analysis

**Fig. 1:**
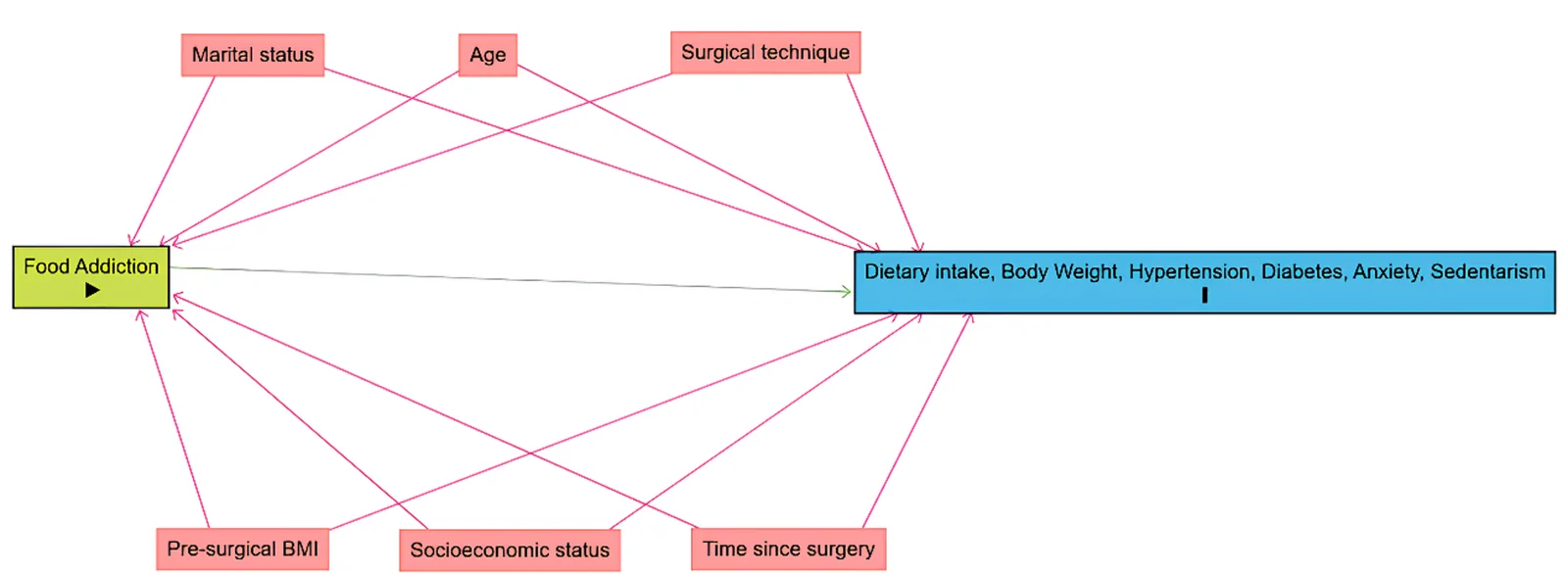
Directed acyclic graph showing the causal pathways between food addiction and the outcomes of interest

All statistical analyses were performed using Jamovi (version 2.3; Jamovi Project, 2025, Sydney, Australia) at the 5% significance level. The comparison of categorical variables between groups with and without FA was performed using Fisher’s exact test, and comparisons of continuous variables between independent groups were conducted using the t-test or Welch’s test. In multivariable analyses, Poisson models with robust variance adjustment were used when the outcomes were dichotomous to estimate prevalence ratios (PRs). For continuous outcomes, an Analysis of Covariance (ANCOVA) was used, with FA presence/absence as the fixed factor. Each outcome was run separately in its own model, which in turn was adjusted for marital status, surgical technique, participants’ age, time since surgery, economic scale score, and pre-surgical BMI. The relationships among these variables and the decision to include them in the model were drawn from a directed acyclic graph (Fig. 1). When the outcome involved dietary intake (NOVA Score or SISVAN markers), the questionnaire response date (weekend or not) was also added as a covariate. Considering that BMI values (pre-surgical, minimum post-operative, and current) for each individual were obtained retrospectively at a single time point, a mixed ANOVA (with one independent factor, the presence of FA, and one dependent factor, the time point of weight measurement) was applied to compare the different FA conditions over time.

## Results

**Fig. 2:**
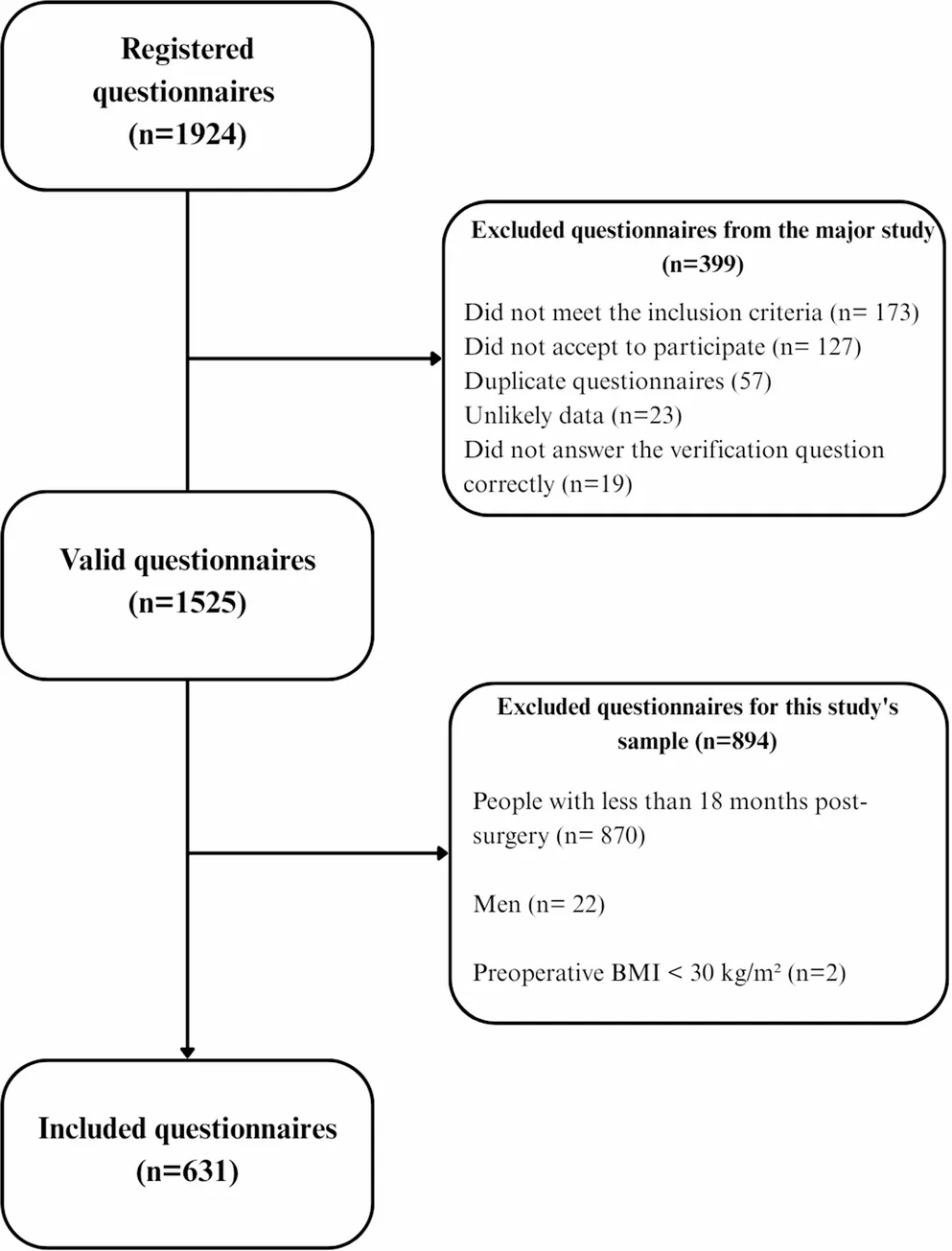
Flowchart of sample selection

The flow diagram of participant inclusion leading to the final sample of 631 participants is shown in Fig. 2. Of the women included in the sample, 124 (19.7%) were classified as having FA. In terms of severity of the construct, 57 participants (9.0%) had mild to moderate FA, while 67 (10.6%) had severe FA. Other sociodemographic and clinical characteristics of the sample, along with anthropometric data, are presented in Table 1.

**Table 1:** Sociodemographic and clinical characteristics of the sample (*n* = 631)

	Total (*n* = 631)		With Food Addiction (*n* = 124)		Without Food Addiction (*n* = 507)		*p*-value
	Mean	%	Mean	SD	Mean	SD	
Age (years)	40.7	8.14	39.34	8.88	41.03	7.93	0.038
Height (m)	1.63	0.06	1.63	0.068	1.63	0.06	0.853
Post MBS time (months)	79.81	59.88	94.82	65.75	76.14	57.84	0.002
Pre-surgical weight (kg)	114.4	15.67	116.13	17.55	113.98	15.17	0.170
Excess Weight (pre) (kg)	47.49	14.26	49.29	15.79	47.05	13.84	0.116
Current weight (kg)	76.71	15.26	84.71	17.48	74.76	14.00	0.001¹
Ideal weight (kg)	66.91	5.4	66.84	5.53	66.93	5.38	0.865
	Total (*n* = 631)		With Food Addiction (*n* = 124)		Without Food Addiction (*n* = 507)		p-value
	**n**	**%**	**n**	**%**	**n**	**%**	
Surgical technique							0.889
RYGB	536	84.9	105	84.7	431	85	
Sleeve gastric	95	15.1	19	15.3	76	15	
Economic class							0.223
A	51	8.1	6	4.8	45	8.9	
B1	130	20.6	23	18.5	107	21.1	
B2	255	40.4	50	40.3	205	40.4	
C1	140	22.2	37	29.8	103	20.3	
C2	48	8.1	7	5.6	41	8.1	
D-E	7	1.1	1	0.8	6	1.2	

*RYGB* roux-en-y gastric bypass, *MBS* metabolic and bariatric surgery

Notably, the percentage of excess weight lost and the %TWL were lower in participants with FA than in those without FA (*P* < 0.001 for both). Recurrent weight gain was greater in the FA group (30.25 kg [27.41–33.10]) than in the non-FA group (18.38 kg [16.99–19.77]; *p* < 0.001). Regarding dietary intake markers, the NOVA score was significantly higher in the FA group (4.37 [3.96–4.79]) than in the non-FA group (3.26 [3.06–3.46]; *p* < 0.001) (Table 2). Participants with FA reported more frequent consumption of sweets and candies, sugar-sweetened beverages, hamburgers and processed meats, instant noodles, savory snacks, and savory crackers (Table 3). Additionally, hypertension and generalized anxiety disorder were more prevalent in this group, while engagement in physical activity was lower. (Table 3).

**Table 2:** Adjusted multivariable models comparing groups with and without food addition for body weight and ultra-processed foods consumption outcomes

Variables	With Food Addiction (*n* = 124)		Without Food Addiction (*n* = 507)		*p*-value¹
	Mean	CI 95%	Mean	CI 95%	
NOVA score	4.37	[3.96; 4.78]	3.26^a^	[3.06; 3.46]	< 0.001
Excess weight lost (%)	98.75	[95.41; 102.08]	104.03^a^	[102.40; 105.66]	0.006
Recurrent weight gain (Kg)	30.25	[27.41; 33.10]	18.38^a^	[16.99;19.77]	< 0.001
Total weight lost (%)	27.47	[25.81; 29.13]	34.13^a^	[33.32; 34.94]	< 0.001

¹p-value for covariance analysis adjusted for the following variables: age, time since surgery, preoperative BMI, socioeconomic status – CCEB score, marital status, and surgical technique. The variable “response on the weekend” was included as an adjustment only in the models referring to the Nova Score. The means presented refer to the marginal means estimated with the model

*CI* Confidence Interval

**Table 3:** Comparison of clinical outcomes and food consumption between groups with and without food addiction

Variables	With food addiction (*n* = 124)		Without food addiction (*n* = 507)					
	*n*	%	*n*	%	*p*-value¹	PR	CI95%	*p*-value¹
Food consumption outcomes								
Fresh fruits	67	54.0	310	61.1	0.154	0.90	[0.75; 1.07]	0.256
Vegetables	83	66.9	377	74.4	0.114	0.90	[0.78; 1.03]	0.143
Beans	69	55.6	289	57.0	0.84	0.96	[0.81; 1.15]	0.716
Sweets and treats	69	55.6	180	35.5	< 0.001	1.58	[1.29; 1.94]	< 0.001
Artificially sweetened drinks	65	52.4	183	36.1	0.001	1.52	[1.24; 1.87]	< 0.001
Hamburger and/or sausages	53	42.7	145	28.6	0.003	1.52	[1.18; 1.95]	< 0.001
Instant noodles. packaged snacks. and/or salty cookies	31	25	80	15.8	0.018	1.66	[1.14; 2.42]	0.008
Clinical outcomes								
Hypertension diagnosis	16	12.9	38	7.5	0.071	1.90	[1.08; 3.35]	0.026
Diabetes diagnosis	6	4.8	11	2.2	0.119	2.68	[0.98; 7.3]	0.054
GAD diagnosis	86	69.4	165	32.5	< 0.001	2.02	[1.69; 2.42]	< 0.001
Physical activity practice	48	38.7	301	59.4	< 0.001	0.65	[0.52; 0.82]	< 0.001

¹P-value for covariance analysis adjusted for the following variables: age, time since surgery, preoperative BMI, socioeconomic status – CCEB score, marital status, and surgical technique

*PR* Prevalence ratio, *CI* Confidence Interval, *GAD* Generalized Anxiety Disorder

**Fig. 3:**
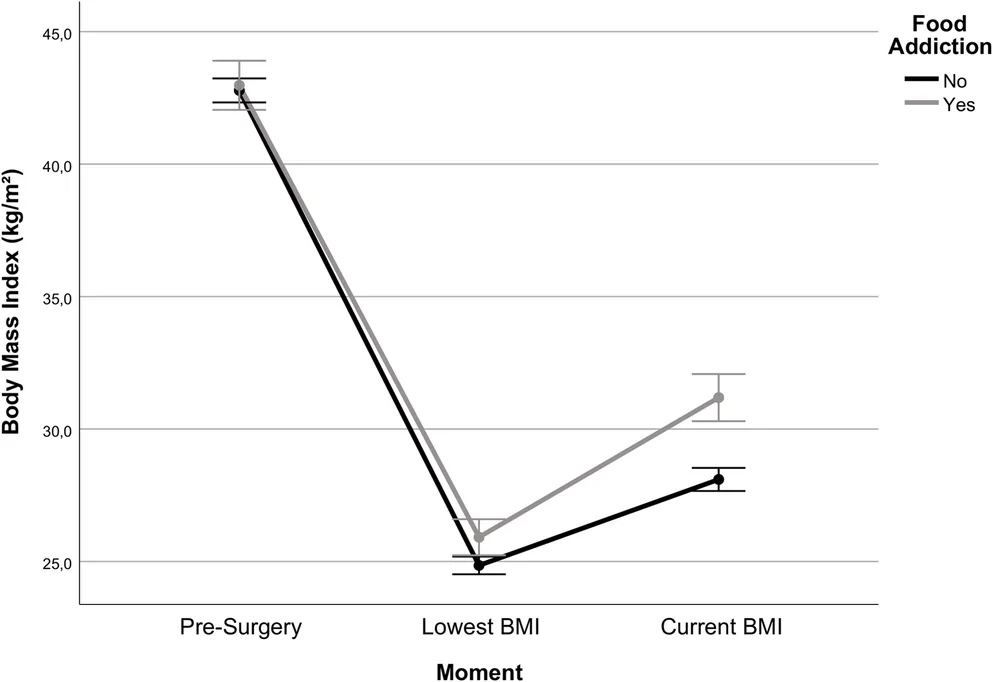
Adjusted mean Body Mass Index (BMI) at three time points (pre-surgery, lowest post-surgical BMI, and current BMI) stratified by food addiction status

**Fig. 4:**
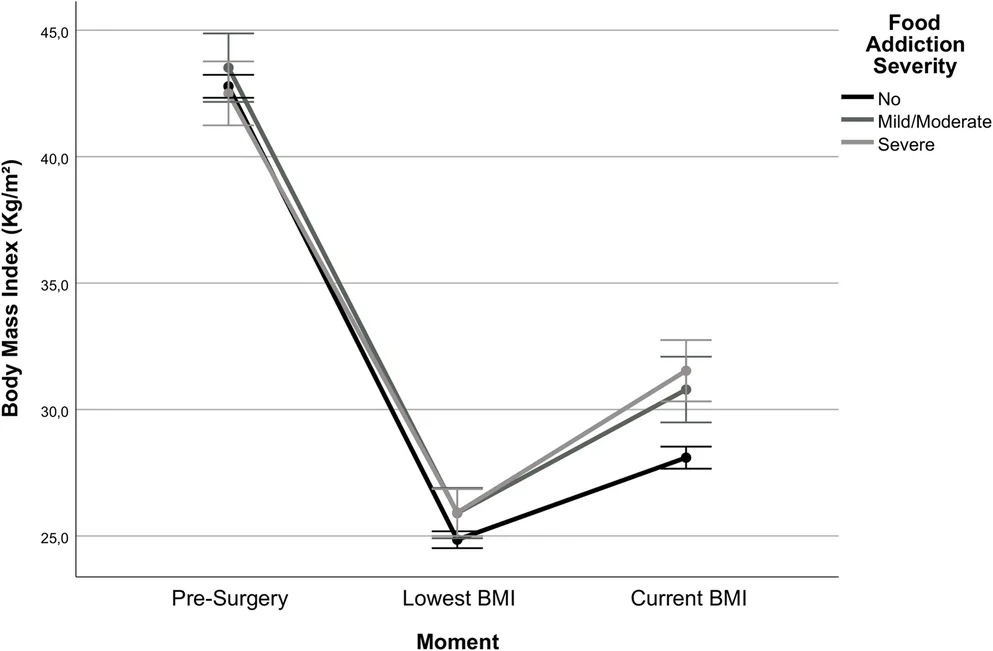
Adjusted mean Body Mass Index (BMI) at three time points (pre-surgery, lowest post-surgical BMI, and current BMI) stratified by food addiction severity (none, mild/moderate, severe)

The lowest BMI achieved after surgery was also significantly higher in participants with FA (25.88 vs. 24.86 kg/m²; *p* < 0.001) (Table 4). Repeated-measures analyses confirmed these differences: participants with FA maintained higher current BMI and lower post-surgery BMI after adjustment (Fig. 3; Table 3). When stratified by FA severity, both current and post-surgical BMI increased progressively with severity (Table 5; Fig. 4).

**Table 4:** Estimated marginal means of body mass index at the preoperative, lowest, and current moment by food addiction diagnosis

Food addiction status	Pre-surgery BMI		Lowest BMI		Current BMI		p-value¹
	Mean	CI 95%	Mean	CI 95%	Mean	CI 95%	
With Food Addiction (*n* = 124)	42.95	[42.03; 43.88]	25.88	[25.19; 26.56]	31.14	[30.25; 32.03]	< 0.001
Without Food Addiction (*n* = 507)	42.78	[42.33; 43.24]	24.86	[24.53; 25.19]	28.10	[27.67; 28.54]	

¹P-value for covariance analysis adjusted for the following variables: age, time since surgery, preoperative BMI, socioeconomic status – CCEB score, marital status, and surgical technique

*BMI* body mass index, *CI* Confidence Interval

**Table 5:** Estimated marginal means of preoperative, lower, and current body mass index according to the severity of food addiction

Food Addiction status	Pre-surgery BMI		Lowest BMI		Current BMI		p-value¹
	Mean	CI 95%	Mean	CI 95%	Mean	CI 95%	
No Food Addiction	42.79	[42.33; 43.24]	24.86	[24.53; 25.19]	28.11	[27.67; 28.54]	< 0.001
Light to moderate	43.49	[42.14; 44.85]	25.83	[24.83; 26.83]	30.71	[29.41; 32.01]	
Severe	42.49	[41.23; 43.74]	25.92	[24.99; 26.85]	31.52	[30.31; 32.73]	

¹P-value for covariance analysis adjusted for the following variables: age, time since surgery, preoperative BMI, socioeconomic status – CCEB score, marital status, and surgical technique

*BMI* body mass index, *CI* Confidence Interval

## Discussion

Our study investigated the association between FA and clinical, behavioral, dietary, and anthropometric variables within a single sample of post-MBS patients. Individuals with FA after MBS exhibited a higher prevalence of generalized anxiety disorder, hypertension, and sedentary behavior, along with greater consumption of UPFs, including sugar-sweetened beverages, hamburgers and processed meats, instant noodles, savory snacks, and savory crackers. They also had lower %TWL and higher weight and BMI over time, observed alongside differences in anthropometric and behavioral outcomes, which may reflect coexisting eating behaviors and emotional factors rather than a causal effect on MBS effectiveness. The prevalence of FA in this study (19.7%) aligns with findings conducted by Praxedes et al. [7], who reported a prevalence of 32% in the preoperatively and 15% in the postoperatively across 40 studies. These data reinforce the relevance of ongoing monitoring of eating behaviors in individuals undergoing MBS.

Considering the findings of the present study, FA after MBS was associated with differences in cardiometabolic and behavioral indicators. Individuals with FA exhibited a higher frequency of self-reported hypertension, sedentary behavior, and more frequent consumption of UPF, and lower %TWL over time. These findings are consistent with previous literature indicating that FA is associated with less favorable weight-related trajectories and distinct behavioral profiles in post-bariatric populations [9].

Previous studies have observed associations between FA, higher UPF consumption, and type 2 diabetes mellitus. FA has been observed in association with type 2 diabetes mellitus Horsager et al. [21], supported by systematic reviews and meta-analyses, which aimed to determine the prevalence of FA in individuals with type 2 diabetes [22], and in specific groups, such as Brazilian women in socioeconomically disadvantaged contexts [23]. Although the cross-sectional design of the present study does not allow assessment of remission or persistence of type 2 diabetes mellitus and systemic arterial hypertension after surgery, these findings indicate that FA may coexist with distinct metabolic and behavioral profiles in the postoperative period, emphasizing the need of ongoing multidisciplinary follow-up after MBS.

Regarding the anthropometric variables of the individuals, the findings align with the results of Mousavi et al. [8], who observed that individuals with FA, two years after vertical gastrectomy, presented with lower excess weight loss, a lower %TWL, and a greater loss of fat-free mass compared to those without FA. In the present study, it was also observed that physical activity was significantly less frequent among individuals with FA, which were observed alongside lower engagement in physical activity and differences in weight-related indicators. Amundsen et al. [24] reinforce that postoperative physical training leads to greater weight and fat loss, increased muscle strength, and increased VO2max, thereby assisting in the weight-loss process and maintaining the lost weight.

Regarding dietary habits, individuals with FA consumed UPF more frequently. Despite the metabolic and endocrine effects associated with MBS, Livingstone et al. [25] demonstrated that, in the postoperative period, patients exhibit quantitative dietary changes while continuing to consume hyperpalatable and UPFs. In this sense, nutritional patterns that reduce intake of UPFs and increase consumption of fresh and minimally processed foods may represent an important tool for maintaining the nutritional quality of the diet and achieving long-term results in this population [26]. Dietary findings may be linked to the observed association between the presence of FA and the presence of generalized anxiety disorder. Cassin et al. [12] identified that those patients after MBS with FA also presented significant levels of anxiety symptoms and difficulty with body image, reinforcing the importance of psychological follow-up in the postoperative period. Anxiety has been frequently associated with FA. Schulte et al. [16] showed that individuals with anxiety tend to resort to the consumption of hyperpalatable foods as a strategy for immediate emotional support, reinforcing the symptoms of possible food dependencies. Physiologically, this relationship can be elucidated through neurobiological mechanisms that activate the dopaminergic system and brain reward circuits, which are stimulated by both the ingestion of foods rich in saturated fat, refined sugar, and salt, as well as by the visualization of such foods [5]. These findings indicate that anxiety, which is more frequent in FA, coexists with maladaptive eating patterns, affecting the weight trajectory and psychological well-being of post-bariatric individuals.

Although MBS may alter taste perception and food preferences, potentially reducing the rewarding properties of hyperpalatable foods, some patients continue to exhibit FA symptoms. Changes in taste perception and hedonic responses after MBS procedures have been widely documented, with many patients reporting alterations in the intensity and desirability of sweet, fatty, and savory tastes following surgery. Systematic reviews indicate that taste sensitivity and hedonic responses to sweet and high-fat stimuli may change after surgery, which could contribute to shifts in dietary behaviors and preferences [27].

Studies also show that a substantial proportion of post-MBS patients experience changes in appetite, taste, and smell, as well as altered cravings for specific foods. For example, many individuals report increased sensitivity to sweet taste or diminished preference for previously favored high-energy foods after surgery [28, 29]. These changes may relate to alterations in gut hormone signaling, neural reward pathways, and sensory processing following surgical modifications of the gastrointestinal tract [30].

Moreover, functional studies suggest that MBS may modulate reward-related neural circuits, including dopaminergic pathways implicated in the motivation to eat and in the hedonic value of food. Such neurobiological alterations have been hypothesized to contribute to reduced cravings and changes in food preferences in some patients, yet they do not universally eliminate maladaptive eating tendencies [31].

Recent evidence indicates that MBS can be effective in reducing FA symptoms in many individuals [32], but the persistence of FA in a subset of patients highlights that physiological changes in taste and preference alone do not fully account for complex eating behaviors. Psychological and behavioral factors…were observed alongside maladaptive eating patterns even when sensory reward responses are altered, indicating that, beyond physiological adaptations, central behavioral and affective mechanisms play a key role in the persistence of problematic eating behaviors after surgery.

This study has some limitations. Recruitment through online surveys using convenience and snowball sampling may have introduced selection bias, favoring participation from individuals with greater interest in eating or weight-related issues and with better access to, or familiarity with, digital platforms. In addition, self-reported anthropometric data can be considered another limitation. The dietary consumption markers of the SISVAN and the NOVA-UPF screener, although validated for the Brazilian population, are qualitative instruments assessing intake on the day preceding data collection. They do not capture portion sizes or habitual dietary intake. A single 24-hour assessment does not represent usual intake and may result in underestimation or exposure misclassification due to substantial intra-individual day-to-day variability. Consequently, associations with weight, BMI, and other outcomes may be attenuated or biased. Furthermore, although mYFAS-2.0 is DSM-5–anchored and widely used, its formal psychometric validation, specifically in post-bariatric samples, remains limited. Consequently, some degree of misclassification of FA status cannot be ruled out, which could have attenuated the magnitude of the observed associations, although most likely non-differential. In addition, our study did not assess other eating disorders among participants. Other studies should focus on the impact of different eating disorders on the outcomes of the MBS. Another limitation of the study is that information on hypertension and diabetes was collected at a single time point through self-reported medical diagnosis. These data reflect participants’ current status at the time of questionnaire completion and were not verified by clinical or laboratory measurements. Therefore, it is not possible to determine pre-surgical status, postoperative remission, or actual metabolic control, limiting the ability to draw causal inferences regarding the impact of FA on these conditions. Another limitation is that only female participants were included. This limits the generalizability of our findings to male post-MBS populations. Preoperative psychological status was not assessed, as anxiety was measured only at the time of data collection, limiting temporal interpretation of its association with food addiction. Finally, our study is observational and cross-sectional, which is prone to confounding, and no causal inferences may be made. The groups of individuals with and without FA were inherently different, especially regarding key variables that may affect our outcomes, such as “time since surgery”. We used multivariable models to address such imbalances, but residual confounding may remain.

## Conclusion

In conclusion, FA was observed in association with higher anxiety symptoms, higher consumption of UPFs, lower engagement in physical activity, higher current weight, and lower %TWL in women in the post-MBS period compared to without FA. These findings reflect associations rather than causal relationships and underscore the importance of ongoing screening and multidisciplinary follow-up for women after MBS.

## Supplementary Information

Below is the link to the electronic supplementary material.

Supplementary file 1 (DOCX 12.3 KB)

Supplementary file 2 (DOCX 15.4 KB)

## Data Availability

The study protocol entitled “Individuals With Food Addiction After Metabolic and Bariatric Surgery Show Higher Consumption of Ultra-Processed Foods, Sedentary Lifestyle, Anxiety, and Worse Body Weight Trajectories” was approved by the Research Ethics Committee of the Federal University of Alagoas (protocol number: 60233722.7.0000.5013). The study was conducted in accordance with the ethical standards outlined in the 1964 Helsinki Declaration and its subsequent amendments. All participants were informed about the study objectives and procedures through an online consent form displayed on the first page of the virtual questionnaire, and consent was required to proceed with the survey.
