# Correction: Individuals With Food Addiction After Metabolic And Bariatric Surgery Show Higher Consumption of Ultra-Processed Foods, Sedentary Lifestyle, Anxiety, and Sub-Optimal Body Weight Trajectories

**DOI:** 10.1007/s11695-026-08967-w

**Published:** 2026-10-01

**Authors:** Maria Clara Tavares Farias da Silva, Jennifer Mikaella Ferreira Melo, Natália Gomes da Silva Lopes, André Eduardo da Silva Júnior, Mateus de Lima Macena, Nassib Bezerra Bueno

**Affiliations:** 1 Laboratório de Nutrição e Metabolismo (LANUM), Programa de Pós-Graduação em Nutrição (PPGNUT), Faculdade de Nutrição (FANUT), Universidade Federal de Alagoas (UFAL), AL Maceió, Brazil; 2 Programa de Pós-Graduação em Nutrição, Escola Paulista de Medicina, Universidade Federal de São Paulo, SP São Paulo, Brazil

**Correction: Obesity Surgery (2026) 36:4042–4051**

**https://doi.org/10.1007/s11695-026-08812-0**

The correct name of the first author is Maria Clara Tavares Farias da Silva.

