# Supplementary material for: Individuals With Food Addiction After Metabolic And Bariatric Surgery Show Higher Consumption of Ultra-Processed Foods, Sedentary Lifestyle, Anxiety, and Sub-Optimal Body Weight Trajectories
Source: Obes Surg. 2026 Jun 27;36(8):4042–51. doi: 10.1007/s11695-026-08812-0 (PMC13429533; doi:10.1007/s11695-026-08812-0)
Supplement: Supplementary file 1 — (DOCX 12.3 KB) [file 11695_2026_8812_MOESM1_ESM.docx]

**Table S1.** Strengthening the Reporting of Observational Studies in Epidemiology - STROBE

| **Item** | **Recommendation** | **Page number(s)** |
| --- | --- | --- |
| **Title and abstract** | a. Indicate the study’s design with a commonly used term in the title or the abstract | 1 |
|  | b. Provide in the abstract an informative and balanced summary of what was done and what was found | 1 |
| **Introduction** |  |  |
| Background/rationale | Explain the scientific background and rationale for the investigation being reported | 3 |
| Objectives | State specific objectives, including any prespecified hypotheses | 4 |
| **Methods** |  |  |
| Study design | Present key elements of study design early in the paper | 4 |
| Setting | Describe the setting, locations, and relevant dates, including periods of recruitment, exposure, follow-up, and data collection | 5 |
| Participants | a. Give the eligibility criteria, and the sources and methods of selection of participants. Describe methods of follow-up  Cross-sectional study – Give the eligibility criteria, and the sources and methods of selection of participants. | 4 |
|  | b. For matched studies, give matching criteria and number of exposed and unexposed | - |
| Variables | Clearly define all outcomes, exposures, predictors, **potential confounders**, and effect modifiers. Give diagnostic criteria, if applicable | 6-8 |
| Data sources/measurement | For each variable of interest, give sources of data and details of methods of assessment (measurement). Describe comparability of assessment methods if there is more than one group | 5 |
| Bias | Describe any efforts to address potential sources of bias | 5 |
| Study size | Explain how the study size was arrived at | - |
| Quantitative variables | Explain how quantitative variables were handled in the analyses. If applicable, describe which groupings were chosen and why | 6-8 |
| Statistical methods | a. Describe all statistical methods, including those used to control for confounding | 8 |
|  | b. Describe any methods used to examine subgroups and interactions | 8 |
|  | c. Explain how missing data were addressed | Figure 2 |
|  | d. If applicable, explain how loss to follow-up was addressed | - |
|  | e. Describe any sensitivity analyses | - |
| **Results** |  |  |
| Participants | a. Report numbers of individuals at each stage of study – e.g., numbers potentially eligible, examined for eligibility, confirmed eligible, included in the study, completing follow-up, and analysed | 9 |
|  | b. Give reasons for non-participation at each stage | Figure 2 |
|  | c. Consider use of a flow diagram | Figure 1 |
| Descriptive data | a. Give characteristics of study participants (e.g., demographic, clinical, social) and information on exposures and potential confounders | Table 1 |
|  | b. Indicate number of participants with missing data for each variable of interest | - |
|  | c. Summarise follow-up time (e.g., average and total amount) | - |
| Outcome data | Report numbers of outcome events or summary measures over time | Tables 2-5 |
| Main results | a. Give unadjusted estimates and, if applicable, confounder-adjusted estimates and their precision (e.g., 95% confidence interval). **Make clear which confounders were adjusted for** and why they were included | Tables 2-5 |
|  | b. Report category boundaries when continuous variables were categorized | 9 |
|  | c. If relevant, consider translating estimates of relative risk into absolute risk for a meaningful time period | - |
| Other analyses | Report other analyses done – e.g., analyses of subgroups and interactions, and sensitivity analyses | 9 |
| **Discussion** |  |  |
| Key results | Summarise key results with reference to study objectives | 10-11 |
| Limitations | Discuss limitations of the study, taking into account sources of potential bias or imprecision. Discuss both direction and magnitude of any potential bias | 10-11 |
| Interpretation | Give a cautious overall interpretation of results considering objectives, limitations, multiplicity of analyses, results from similar studies, and other relevant evidence | 10-11 |
| Generalisability | Discuss the generalisability (external validity) of the study results | 10-11 |
| **Other information** |  |  |
| Funding | Give the source of funding and the role of funders for the present study and, if applicable, for the original study on which the present article is based | 12 |
